# De Novo Endotoxin-Induced Production of Antibodies against the Bile Salt Export Pump Associated with Bacterial Infection following Major Hepatectomy

**DOI:** 10.1155/2018/6197152

**Published:** 2018-04-23

**Authors:** Kun-Ming Chan, Chih-Hsien Cheng, Tsung-Han Wu, Chen-Fang Lee, Ting-Jung Wu, Hong-Shiue Chou, Wei-Chen Lee

**Affiliations:** Department of General Surgery, Chang Gung Memorial Hospital at Linkou, Chang Gung University College of Medicine, Taoyuan, Taiwan

## Abstract

**Background:**

Clinically severe infection-related inflammation after major liver resection may cause hyperbilirubinemia. This study aims to clarify the impact of bacterial infection and endotoxins on the hepatobiliary transporter system and to explore possible mechanisms of endotoxin-related postoperative hyperbilirubinemia.

**Method:**

Mice that underwent major hepatectomy with removal of at least 70% of liver volume were exposed to lipopolysaccharide (LPS) at different dosages. Subsequently, hepatobiliary transporter compounds related to bile salt excretion were further investigated.

**Results:**

The expression of genes related to hepatobiliary transporter compounds was not significantly different in the liver tissue of mice after major hepatectomy and LPS exposure. However, bile salt export pump (BSEP) protein expression within the liver tissue of mice treated with LPS after major hepatectomy was relatively weaker and was even further reduced in the high-dose LPS group. The formation of antibodies against the BSEP in response to endotoxin exposure was also detected.

**Conclusion:**

This study illustrates a possible mechanism whereby the dysfunction of hepatobiliary transporter systems caused by endotoxin-induced autoantibodies may be involved in the development of postoperative jaundice associated with bacterial infection after major hepatectomy.

## 1. Introduction

Liver resection for the removal of tumours is considered best practice for treating hepatic malignancy and can nowadays be performed safely with low mortality and morbidity [1, 2]. However, up to 75% of patients that undergo liver surgery have abnormal postoperative liver function tests results, which may be accompanied by jaundice in some patients [3–6]. Clinically, severe postoperative jaundice is rare in patients with no preexisting underlying liver disease and a sufficient remnant liver volume. Nonetheless, severe infection-related inflammation may result in hyperbilirubinemia after liver resection, which might be associated with higher rates of morbidity and mortality [7, 8]. Hence, it is of great importance to identify potential mechanisms for the development of hyperbilirubinemia related to infection-associated inflammation after liver resection.

The reasons for the development of postoperative jaundice remain unknown; however, jaundice is known to be caused by defects in the hepatobiliary transporter systems that are important for normal bile salt excretion, leading to cholestasis [9–11]. The bile salt export pump (BSEP) is a crucial hepatobiliary transporter system located on the canalicular membrane of hepatocytes, which is involved in the excretion of bile salts from the liver into the bile [12]. Dysfunction of the BSEP, in particular, is considered a factor associated with increased susceptibility for liver injury [13, 14]. However, little is known about the relationship between postoperative bacterial infection and the hepatobiliary transporter system in the pathogenesis of hyperbilirubinemia after liver resection. Therefore, the present study aims to clarify the impact of bacterial infection and endotoxins on the hepatobiliary transporter system and to explore the possible mechanism of endotoxin-related postoperative cholestasis.

## 2. Materials and Methods

### 2.1. Animal Experiments

Six- to 8-week-old male C57BL/10J (B10) mice were purchased from the National Laboratory Animal Center, Taipei Center, Taiwan, and maintained in the pathogen-free facility of Change-Gung Memorial Hospital in Linkou. All experimental procedures for these mice were approved by the Animal Care and Use Committee of Chang Gung Memorial Hospital. The use of animals was also in accordance with the Animal Protection Law of the Council of Agriculture, Executive Yuan, Taiwan, and the guidelines in the Guide for the Care and Use of Laboratory Animals from the Institute of Laboratory Animal Resources of the National Research Council, USA. Partial hepatectomy was performed by removing certain hepatic lobes, as previously described, and major hepatectomy was defined as the removal of at least 70% of the liver volume [15]. Lipopolysaccharide (LPS; Sigma, St. Louis, MO, USA) was dissolved in sterile sodium chloride (9 g/L) and injected intravenously at the experimental dosage via the infraorbital venous plexus or tail vein the day following the hepatectomy. Mice were sacrificed at different time points for the collection of specimens. Blood was either collected by cardiac puncture or drawn from the inferior vena cava. The liver was removed and immediately frozen in liquid nitrogen for mRNA and protein analyses, in addition to histological examination. All experiments were repeated at least three times for each group.

### 2.2. RNA and Protein Expression Analyses

Total RNA was extracted from liver tissue using the RNeasy Mini Kit (Qiagen Sciences, Valencia, CA, USA) according to the manufacturer's protocol. First-strand cDNA was synthesised from 2 *μ*g of total RNA using the SuperScript first-strand synthesis system (Invitrogen, Carlsbad, CA, USA). Next, gene expression as indicated by mRNA levels was analysed by real-time polymerase chain reaction (PCR), in which the target cDNA was amplified by AmpliTaq DNA polymerase (Applied Biosystems, Foster City, CA, USA). The thermal cycling parameters for PCR were as follows: preamplification denaturation for 10 min at 95°C; 45–50 cycles of 95°C for 45 s, 54–65°C annealing for 45 s, and extension at 72°C for 45–90 s; then a final extension at 72°C for 8 min. Beta-actin was used as the loading control. The primer sequences of RNA used in this study are listed in Table 1.

Protein expression was assessed by western blotting. Total protein was extracted from liver tissue in radioimmunoprecipitation assay buffer (RIPA buffer; Pierce, Rockford, IL, USA) supplemented with complete protease inhibitor cocktail (Roche Applied Science, Indianapolis, IN, USA). The extracted protein was loaded onto a 12% sodium dodecyl sulfate-polyacrylamide (SDS-PAGE) gel for electrophoresis; then proteins were transferred from the gels onto nitrocellulose membranes (Amersham Biosciences, Piscataway, NJ, USA). The membranes were blocked with 5% milk in 0.1% Tween-20 in Tris-buffered saline and incubated with primary antibodies, including an anti-BSEP antibody (LifeSpan Biosciences, Seattle, WA, USA), anti-MRP2 (multidrug resistance-associated protein 2) antibody (Cell Signaling Technology, Beverly, MA, USA), anti-Ntcp (Na^+^-dependent taurocholate transporter) antibody, and anti-*β*-actin antibody (Millipore, Billerica, MA, USA). Protein patterns were measured with horseradish peroxidase-conjugated secondary antibodies (AB Science, Taipei, Taiwan) and electrochemiluminescence (Amersham Pharmacia, Milan, Italy).

An indirect immunoblot assay was performed to test the presence of BSEP antibodies in the sera after hepatectomy as previously described by Lin et al. [16]. Briefly, membrane protein lysate (40 *μ*g) from normal liver tissue was subjected to western blot analysis. Proteins were separated by 12% SDS-PAGE, transferred onto nitrocellulose membranes, and then cut into strips. Strips were probed with sera obtained from experimental mice or with a commercial anti-BSEP antibody (positive control). The antibody-protein complexes were then detected with horseradish peroxidase-conjugated goat anti-mouse IgM secondary antibodies (AB Science, Taipei, Taiwan) and the signal was developed with electrochemiluminescence (Amersham Pharmacia, Milan, Italy). (Supplemental Figure 1)

### 2.3. Immunopathological Examination

Sera levels of immunoglobulin M (IgM) were measured using a mouse IgM ELISA kit according to the manufacturer's instructions. Immunohistochemical staining was performed on paraffin-embedded sections of the liver specimens according to the manufacturer's instructions. Immunohistochemical staining was performed using polyclonal antibodies against BSEP (LifeSpan Biosciences, Seattle, WA, USA). Immunofluorescence staining was performed by staining cryosections with DyLight 488 (Bethyl Laboratories, Montgomery, TX, USA) conjugated anti-BSEP antibodies and DyLight 594 (Bethyl Laboratories, Montgomery, TX, USA) conjugated anti-IgM antibodies. Nuclei counterstaining was performed with TO-PRO3 (Invitrogen, Carlsbad, CA, USA) according to the manufacturer's instructions. The immunofluorescence-stained sections were subsequently visualised under a Bio-Rad Radiance 2100 mp multiphoton/confocal microscope (Bio-Rad, Hercules, CA, USA), and images were captured and analysed using Image J version 1.37 software.

## 3. Results

### 3.1. Impact of Liver Resection Extent and Endotoxin on Survival

**Figure 1:**
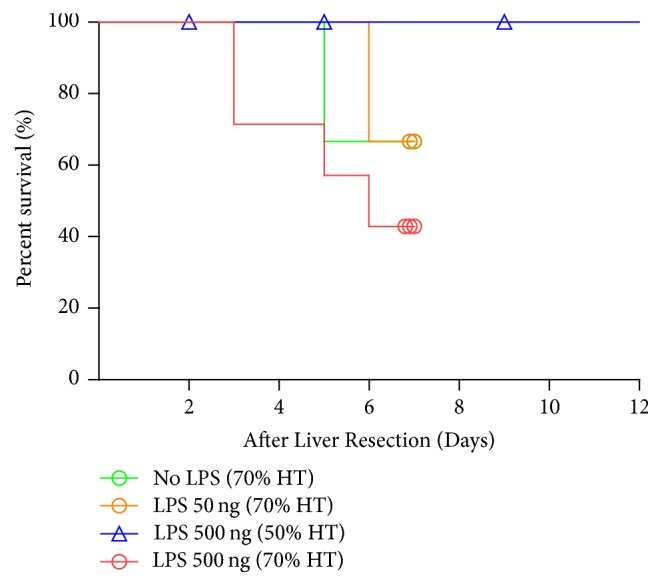
*Kaplan-Meier survival of mice that underwent hepatectomy (HT)*. Mice that underwent 70% HT that were subsequently treated with high-dose lipopolysaccharide (LPS) had higher postoperative mortality than the other groups.

To determine the influence of bacterial infection and extent of liver resection on the outcome of mice after hepatectomy, mice were subjected to either 50% or 70% liver resection followed by administration of LPS on postoperative day 1. The results showed that mice tolerated the 50% liver resection well, even after administration of LPS. However, a few mice died after the 70% liver resection, and the mortality rate was higher with increasing dosages of LPS (Figure 1). Administration of a higher dosage of LPS was associated with a poorer survival curve compared to the other groups. These results indicate that the postoperative bacterial infection does indeed affect the outcome of major liver resection, and the severity of infection in regard to the LPS dosage could also influence survival.

### 3.2. Expression of Hepatobiliary Transporters Related to Bile Salt Excretion after Hepatectomy

**Figure 2:**
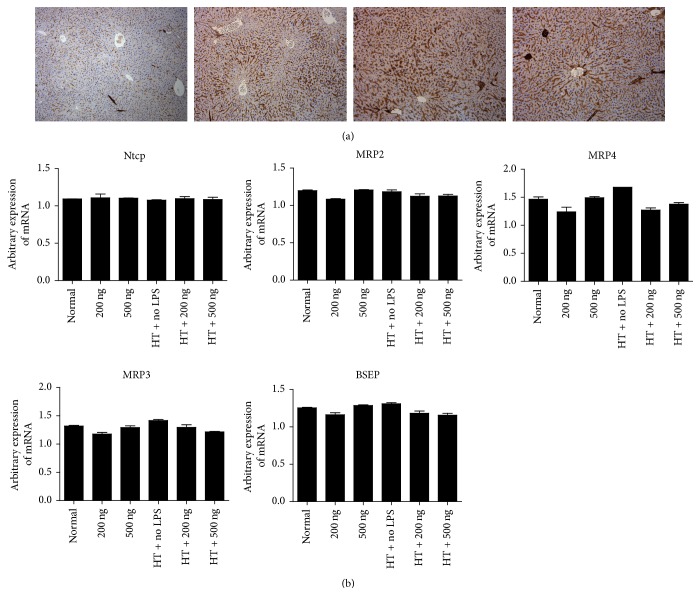
*Examination of hepatobiliary transporter compounds*. (a) Immunohistochemical staining of bile salt export pump (BSEP) in liver tissue showed no difference, even when compared with normal control liver (20x). From left to right: normal liver tissue without hepatectomy and LPS exposure, liver tissue with hepatectomy plus LPS 200 ng at postoperative day 4, liver tissue with hepatectomy plus LPS 500 ng at postoperative day 2, and liver tissue with hepatectomy plus 500 ng LPS at postoperative day 4. (b) The mRNA expression levels of MRP2, MRP3, MRP4, BSEP, and Ntcp were examined, and no significant differences were observed between groups.

Previous studies have shown that numerous hepatobiliary transporter compounds including Ntcp, BSEP, and multidrug resistance-associated proteins (MRP2, MRP3, and MRP4) play important roles in the bile salt excretion of hepatocytes [10, 17]. Therefore, LPS was administered after major hepatectomy to examine the impact of bacterial endotoxin on the hepatobiliary transporter during the period of liver regeneration. Hepatobiliary transporter compounds related to bile salt excretion were analysed in the liver tissue, and the expression of hepatobiliary transporters including MRP2, MRP3, MRP4, Ntcp, and BSEP was found to be similar in the control mice and the LPS-treated mice after hepatectomy in terms of immunohistochemical staining and mRNA levels. The immunohistochemical staining of BSEP expression showed no difference in the liver tissue of mice that were subjected to hepatectomy when compared to control livers (Figure 2(a)). The gene expression of mRNA for MRP2, MRP3, MRP4, Ntcp, and BSEP was also examined, and no significant differences were observed (Figure 2(b)).

**Figure 3:**
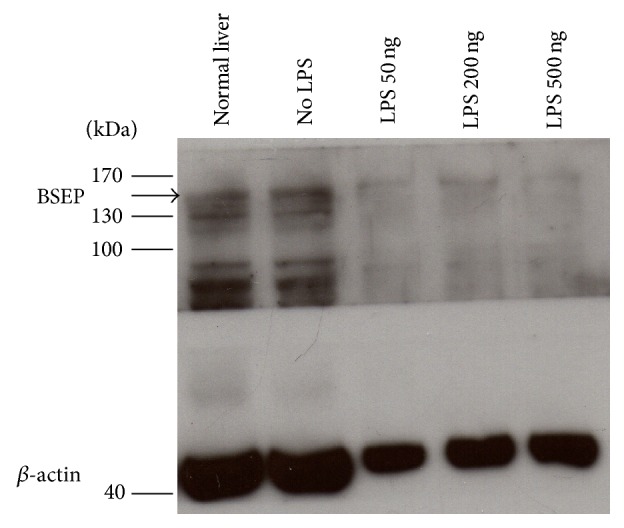
*Bile salt export pump (BSEP) protein expression on the hepatocyte membrane*. Western blot of BSEP protein expression in liver tissue at postoperative day 4 showed a relative decrease in BSEP expression in the hepatocyte membrane of mice that had undergone 70% hepatectomy and subsequently treated with lipopolysaccharide (LPS). Arrow indicates BSEP protein expression (146 kDa).

Because the BSEP is the main transport pathway for bile efflux, we hypothesized that BSEP may be destroyed or dysfunctional as a result of endotoxin administration during the course of liver regeneration after major hepatectomy. Therefore, we tested the expression of the BSEP protein within the liver tissue of mice treated with LPS after major hepatectomy, and the results showed that BSEP protein expression was relatively lower in groups treated with LPS and was even further reduced as the dosage increased (Figure 3). These results suggest that bacterial endotoxin might lead to impaired BSEP protein expression and reduced functional capacity of BSEP in the hepatocytes during the initial period of liver regeneration after major hepatectomy, which might be further reduced by severe bacterial infection.

### 3.3. Identification of Endotoxin-Induced Antibodies after Major Hepatectomy

**Figure 4:**
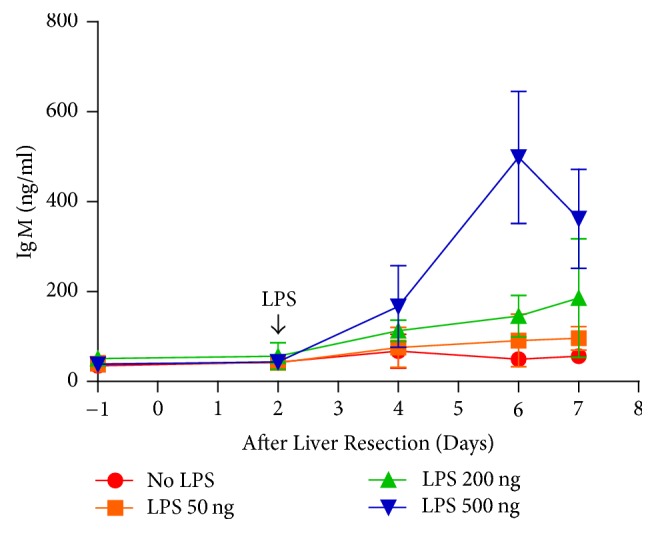
Serum IgM level of mice that underwent 70% hepatectomy followed by lipopolysaccharide (LPS) treatment. The serum IgM level progressively increased after LPS injection and reached the highest level at postoperative day 6. The increase in IgM is correlated with the LPS dosage.

**Figure 5:**
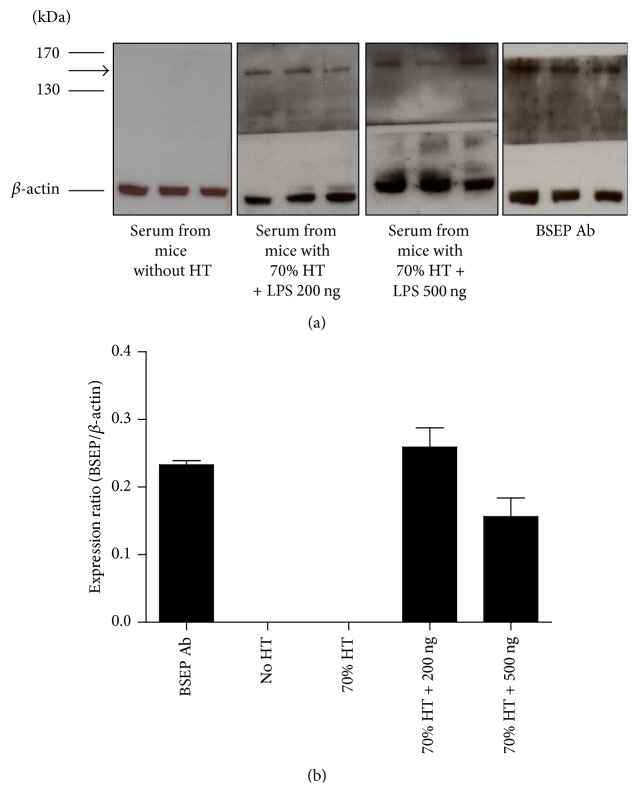
*Indirect immunoblot assay to test the presence of antibodies against bile salt export pump (BSEP) in the sera after hepatectomy (HT)*. (a) Membrane protein lysate from normal liver tissue was subjected to western blot analysis with control anti-BSEP antibodies (BSEP Ab) or sera obtained from experimental mice. Blots with sera as primary antibodies and with peroxidase-labeled anti-mouse IgM as secondary antibodies showed that the presence of BSEP antibodies was detected at postoperative day 6 in mice that had undergone 70% HT and LPS treatment. The experiments were done by triple lanes with the same protein sample from normal liver tissue. Each stripe represented intensity of BSEP antibody within sera from experimental mouse as shown by legend in the left-hand side. (b) Quantification intensity of BSEP antibody expression within sera. Mice that had not undergone HT and mice that had undergone HT without LPS treatment had no detectable BSEP antibodies in sera at the same time point. Arrow indicates BSEP (146 kDa).

The newly formed antibodies in response to exposure to endotoxin were examined. The dynamic expression of IgM in mice subjected to 70% hepatectomy and LPS injection was investigated. The results showed that the serum level of IgM increased gradually after LPS treatment, reaching its highest level at day 4. Additionally, the increments of increased IgM appeared to be correlated with the LPS dosage (Figure 4). Furthermore, an indirect immunoblot assay was performed to test the presence of the BSEP antibody in the sera after hepatectomy. Membrane protein lysate from normal liver tissue which reasonably contains BSEP protein was subjected to western blot for gel electrophoresis of BSEP protein. After electroblotting of nitrocellulose membrane from the gel electrophoresis, anti-BSEP antibodies (positive control) or sera obtained from experimental mice were applied to the nitrocellulose membrane as a primary antibodies for testing the existence of BSEP antibody. Antibodies against BSEP were detectable in the sera obtained from mice that underwent 70% hepatectomy followed by LPS treatment (Figure 5(a)). However, antibodies against BSEP were not detected in the sera from normal mice, nor in control mice that had undergone 70% hepatectomy without LPS treatment (Figure 5(b)). These results suggest that bacterial infection might lead to the production of antibodies specifically against BSEP during the initial period of liver regeneration, as well as impaired BSEP function.

### 3.4. De Novo Production of Antibodies against the Bile Salt Export Pump

**Figure 6:**
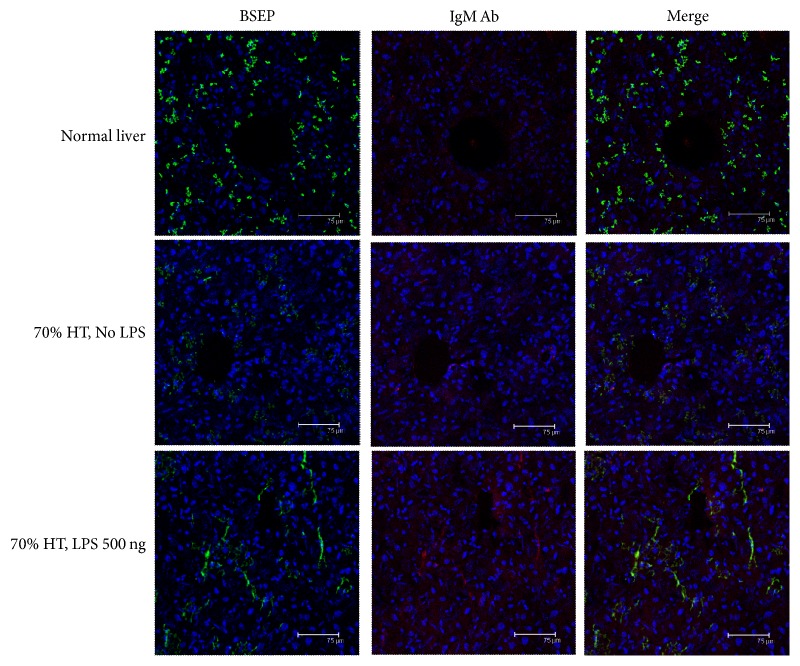
*Immunofluorescence analysis of newly formed antibodies against bile salt export pump (BSEP) protein and IgM antibodies*. Positive staining of both IgM and BSEP was detected in certain areas of the liver tissue of mice that had undergone 70% hepatectomy plus high-dose lipopolysaccharide (LPS) treatment. However, weak or barely detectable double positive for IgM and BSEP was noted in the liver tissue of mice that had not undergone hepatectomy or in those that had undergone 70% hepatectomy without LPS treatment.

To determine whether BSEP was targeted by the newly produced BSEP antibodies, we further examined BSEP and IgM expression within the liver tissue by immunofluorescence staining (Figure 6). Positive staining for both IgM and BSEP was observed in certain areas of liver tissue from mice that had undergone 70% hepatectomy followed by 500 ng LPS treatment, whereas very weak or barely detectable staining for IgM and BSEP was noted in the liver tissue of mice that had not undergone hepatectomy or those that had undergone 70% hepatectomy but were not treated with LPS. These findings, together with the preceding data, suggest that LPS-induced de novo antibody production against BSEP occurred. Therefore, the main transport pathway of bile efflux, BSEP, could potentially be targeted by the LPS-induced antibodies, leading to BSEP dysfunction and hyperbilirubinemia after major hepatectomy.

## 4. Discussion

Clinically severe postoperative bacterial infection may lead to hyperbilirubinemia after partial hepatectomy, related to a higher risk of morbidity and mortality. The reasons for the development of postoperative jaundice remain unknown, but it is known that jaundice can be caused by defects in hepatobiliary transporter systems that are important for normal bile salt excretion. This study intended to characterize factors that may induce posthepatectomy jaundice and liver failure associated with bacterial infection. The results showed that the hepatobiliary transporter system, as indicated by BSEP expression, was mildly decreased under the high-dose LPS endotoxin challenge. Furthermore, de novo anti-BSEP antibodies were detected in posthepatectomy livers after administration of bacterial endotoxin, and antibodies against BSEP were also found in the liver tissue. Therefore, the results of the current study suggest that a possible mechanism may include an interaction between endotoxin and the bile salt transporter system in the liver after major hepatectomy with severe bacterial infection.

The liver is a quiescent organ that has the unique capacity to regulate its growth and mass in both humans and animals. There are many conditions under which a change in liver mass may occur; for example, deficits may result from cell loss caused by chemicals, viruses, or other toxic agents or from surgical removal of liver tissue. Under each of these conditions, quiescent hepatocytes become proliferative and replicate to restore the functional capacity and mass of the liver. The set point for growth regulation is weighted against the balance between the liver mass and body mass. Optimisation of this balance occurs when the liver reaches a state in which the metabolic activity meets the functional requirements of the body. As a result, segmental or lobar resections are applied in clinical settings for the removal of tumours or for living-donor liver transplantation. It has been reported that at least 25% of the future remnant liver volume or at least 30% of standard liver volume should be maintained in order to meet the metabolic demand of the patient after liver resection [18–20]. However, an accurate estimation of the smallest liver volume that meets the functional demands of the body has not yet been established. The higher the ratio of liver volume removed, the higher the risk of postoperative morbidity and mortality in terms of hepatic dysfunction. In line with previous reports, the results of the current study suggest that removal of a greater liver volume would set the host in a vulnerable situation, leading to a higher chance of lethal complications and mortality related to liver dysfunction.

Although the existence of an underlying liver disease prior to liver surgery might cause abnormal postoperative liver function test results, various factors may also be responsible for the development of postoperative jaundice, including intraoperative blood loss with consecutive blood transfusions, medication, sepsis, and oxidative stress, even for a healthy liver. Inflammatory cholestasis has been identified as one the most frequent causes of jaundice in hospitalised patients [21, 22]. Specifically, sepsis-associated cholestasis is frequently observed in critically ill patients, the most common cause of which is the inhibition of hepatobiliary transporter expression and function due to bacterial endotoxins [23, 24]. Although the immunohistochemical staining showed no overt downregulated expression of BSEP within the liver tissue of mice in the groups that received LPS treatment, BSEP protein expression was found to be decreased by LPS treatment in the western blotting analysis. These results indicate that BSEP might be inhibited or functionally impaired by LPS and other bacterial endotoxins.

Bile salt export pumps are exclusively expressed on hepatocytes. There are a few hereditary diseases that cause BSEP deficiency, which may result in severe cholestasis and end-stage liver disease [25]. Moreover, previous studies have shown that anti-BSEP antibodies are induced in certain circumstances or diseases, which can lead to cholestasis [16, 26–28]. Based on the indirect immunoblot assay, BSEP antibodies were detected in the sera of mice that had undergone major hepatectomy followed by LPS treatment, but no antibodies were detected in the sera of control mice or mice that underwent hepatectomy without subsequent LPS treatment. The results suggest that BSEP autoantibodies might be induced by the endotoxin challenge. However, the exact mechanism that triggered the formation of autoantibodies against BSEP remains largely unclear and cannot be explained by the results of the present study.

Usually, many proteins are present in bile, in which proteins are mostly come from plasma through blood to bile transfer by simple diffusion across the semipermeable tight junction of paracellular pathway [29, 30]. However, few proteins such as secretory IgA, IgM, ceruloplasmin, and haemoglobin presumably enter bile as content of intracellular vesicles that fuse with the canalicular membrane [31]. Moreover, immunoglobulin J chain that is a protein component of the antibodies IgM and IgA has previously been identified in bile suggesting that IgA and IgM are capable of entering the canalicular space [30]. The inhibitory effect of BSEP autoantibodies could result from interference with the assembly of microdomains and disturbing the coordinated action of canalicular transporters or potentially direct mechanical occlusion of the BSEP pore by the antibodies [26]. Apart from that, a purified de novo BSEP antibody as well as a cell line transfected with BSEP to test the inhibitory effect of autoantibodies might be able to clarify the pathophysiological mechanism in the future.

Although these antibodies were identified against the BSEP protein, no overt cholestasis was noted in mice that underwent major hepatectomy plus LPS treatment in the current study. The phenomenon may be explained by the quantity of antibody possibly being too low to induce cholestasis, and the antibody titre threshold that could lead to cholestasis or hyperbilirubinemia needs to be confirmed by a more detailed scientific study. Similarly, the mRNA expression of hepatobiliary transporter compounds was unchanged after LPS treatment and might also be related to no overt cholestasis of mice in this study. However, in contrast to study that had showed downregulation of hepatobiliary transporter in inflammatory cholestasis [32, 33], few reports also indicated that some unchanged or even upregulated transport systems such as MRP2 and MRP3 in endotoxin challenged liver tissue [23, 34, 35]. Additionally, species differences regarding expression of hepatobiliary transporter systems might also exist. The animal model of partial hepatectomy through the rodent liver of multilobulated character perhaps is not similar to human hepatectomy that is characterized by relatively more invasive procedure and severe inflammatory reaction.

Taken together, extensive major hepatectomy with a small remnant liver mass is usually very risky, and subsequent development of a severe bacterial infection could result in the hepatic failure or mortality. In general, hyperbilirubinemia and jaundice are the most common presentations of hepatic dysfunction. Although numerous causes may be responsible for the development of postoperative jaundice, the current study illustrates a possible mechanism whereby dysfunction of hepatobiliary transporter systems due to endotoxin-induced autoantibody production may play a role in the development of jaundice after major hepatectomy with concurrent bacterial infection.

## Figures and Tables

**Table 1:** List of RNA primers.

Gene	Forward primer sequence
	Reverse primer sequence
Abcb11 (BSEP)	5′-AGACAGGCAACCCGTCATGGACT-3′ 5′-ACGAACGCCGTCGTTTCCCC-3′
Abcc2 (Mrp2)	5′-TAATGAGGCGCCGTGGGTGAC-3′ 5′-GTCCTGCCCACCACACCGAC-3′
Abcc3 (Mrp3)	5′-GGGCTGCCTTGCCCTGCTAC-3′ 5′-CCGAGGGCCGTCTTGAGCCT-3′
Abcc4 (Mrp4)	5′-CCGAGGTGAAACCCAACCCGC-3′ 5′-CGGGTTGAGCCACCAGAAGAACA-3′
Slc10a1 (Ntcp)	5′-AATCCAAGCTGCAGACGCACC-3′ 5′-GCATCTTCTGTTGCAGCAGCCTT-3′
GAPDH	5′-TGCACCACCAACTGCTTAG-3′ 5′-GATGCAGGGATGATGTTC-3′

BSEP, bile salt export pump; Mrp2, multidrug resistance-associated protein 2; Mrp3, multidrug resistance-associated protein 3; Mrp4, multidrug resistance-associated protein 4; Ntcp, Na^+^-dependent taurocholate transporter; GAPDH, glyceraldehyde-3-phosphate dehydrogenase.

## Supplementary Materials

Supplementary Materials Supplemental Figure 1:* schematic illustration of an indirect immunoblot assay to test the presence of BSEP antibodies in the sera*. Membrane protein lysate from normal liver tissue was subjected to western blot for gel electrophoresis of BSEP protein. After electroblotting of nitrocellulose membrane from the gel electrophoresis, anti-BSEP antibodies (positive control) or sera obtained from experimental mice were applied to the nitrocellulose membrane as the primary antibodies for testing the existence of BSEP antibody. The antibody-protein complexes were then detected with horseradish peroxidase-conjugated goat anti-mouse IgM secondary antibodies, and the signal was developed with electrochemiluminescence as shown in Figure 5(a). The experiment was done by triple lanes with the same protein sample from normal liver tissue. Each stripe represented intensity of BSEP antibody of sera from experimental mouse.
