# Supplementary material for: De Novo Endotoxin-Induced Production of Antibodies against the Bile Salt Export Pump Associated with Bacterial Infection following Major Hepatectomy
Source: Biomed Res Int. 2018 Apr 23;2018:6197152. doi: 10.1155/2018/6197152 (PMC5937615; doi:10.1155/2018/6197152)
Supplement: Supplementary Materials — Supplemental Figure 1: schematic illustration of an indirect immunoblot assay to test the presence of BSEP antibodies in the sera. Membrane protein lysate from normal liver tissue was subjected to western blot for gel electrophoresis of BSEP protein. After electroblotting of nitrocellulose membrane from the gel electrophoresis, anti-BSEP antibodies (positive control) or sera obtained from experimental mice were applied to the nitrocellulose membrane as the primary antibodies for testing the existence of BSEP antibody. The antibody-protein complexes were then detected with horseradish peroxidase-conjugated goat anti-mouse IgM secondary antibodies, and the signal was developed with electrochemiluminescence as shown in Figure 5(a). The experiment was done by triple lanes with the same protein sample from normal liver tissue. Each stripe represented intensity of BSEP antibody of sera from experimental mouse. [file 6197152.f1.pptx]

## Slide 1
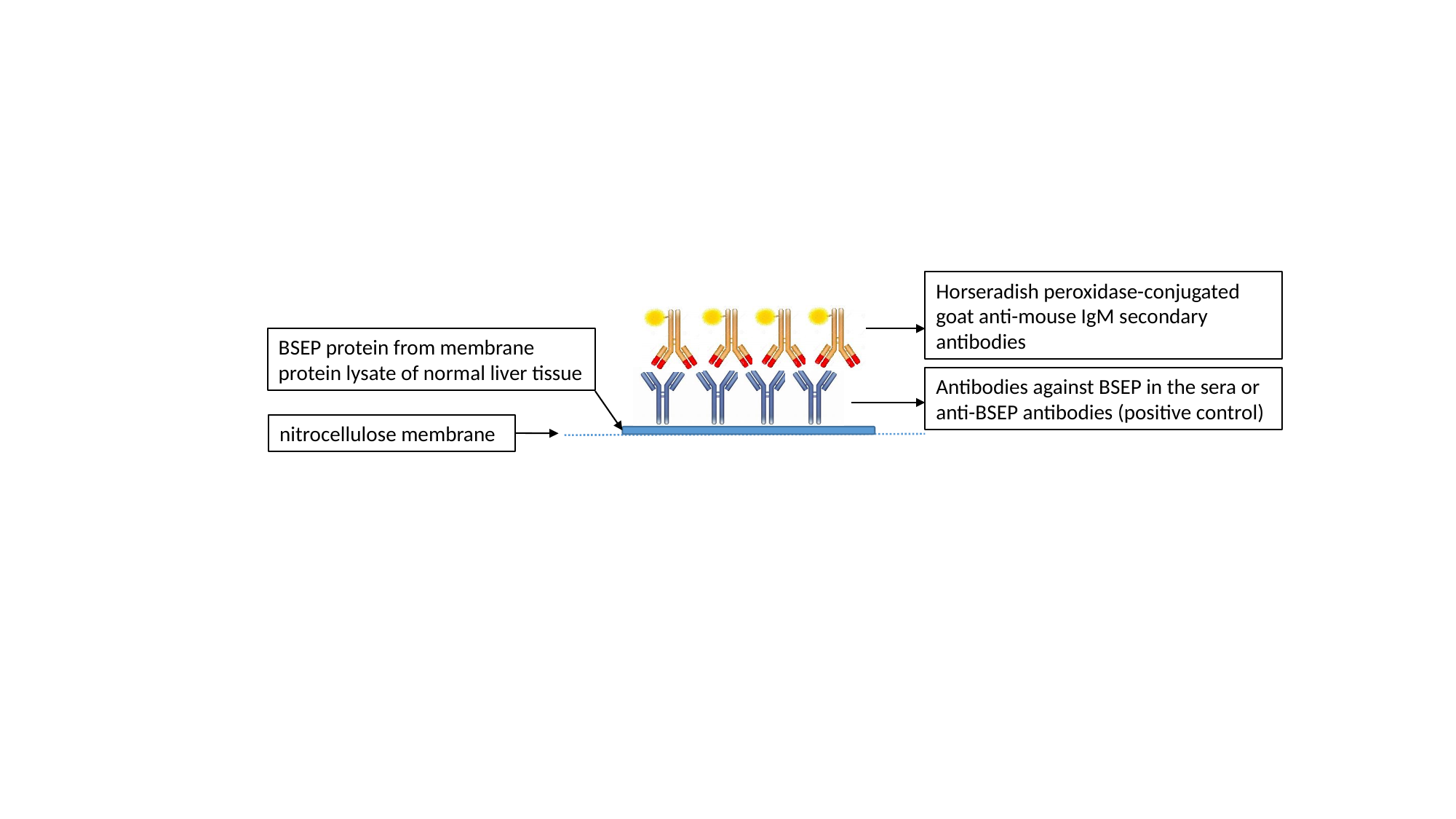

Horseradish peroxidase-conjugated goat anti-mouse IgM secondary antibodies
BSEP protein from membrane protein lysate of normal liver tissue
Antibodies against BSEP in the sera or anti-BSEP antibodies (positive control)
nitrocellulose membrane
